# Cytokinergic IgE Action in Mast Cell Activation

**DOI:** 10.3389/fimmu.2012.00229

**Published:** 2012-08-06

**Authors:** Heather J. Bax, Anthony H. Keeble, Hannah J. Gould

**Affiliations:** ^1^Randall Division of Cell and Molecular Biophysics, King’s College LondonLondon, UK

**Keywords:** allergy, asthma, conformational isomerism, cytokinergic, IgE, mast cell, SPE-7

## Abstract

Some 10 years ago it emerged that at sufficiently high concentrations certain monoclonal mouse IgEs exert previously unsuspected effects on mast cells. Thus they can both promote survival and induce activation of mast cells without the requirement for antigens. This was a wake up call that appears to have been missed (or dismissed) by the majority of immunologists. The structural attributes responsible for the potency of the so-called “highly cytokinergic” or HC IgEs have not yet been determined, but the events that ensue when such IgEs bind to the high-affinity receptor, FcεRI, on mast cells have been thoroughly studied, and are strikingly similar to those engendered by antigens when they form cross-linked complexes with the receptors. We review the evidence for the cytokinergic activity of IgE, and the structural features and known properties of immunoglobulins, and of IgE in particular, most likely to be implicated in the phenomenon. We suggest that IgEs with cytokinergic activity may be generated by local germinal center reactions in the target organs of allergy. We consider also the important implications that the existence of cytokinergic IgE may have for a fuller understanding of adaptive immunity and of the action of IgE in asthma and other diseases.

## Introduction

**Figure 1:**
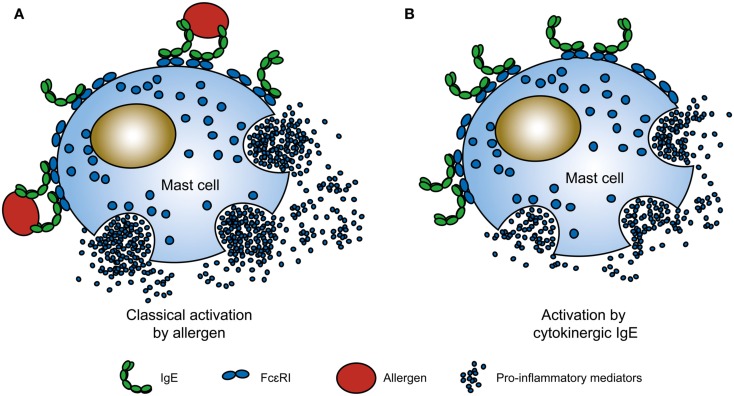
**Comparison between classical allergen and cytokinergic IgE activation of mast cells**. **(A)** In the classical allergic response, multivalent allergens cross-link IgE-FcεRI complexes on the surface of mast cells to trigger mast cell activation, e.g., mast cell degranulation. **(B)** In contrast, the binding of highly cytokinergic (HC) IgE to FcεRI activates mast cells in the absence of antigen. Cytoplasmic α, and the β and γ chains of FcεRI have been omitted for simplicity. See Figure 3 and Section “Mechanisms of Cytokinergic Activity” for possible mechanisms for cytokinergic IgE cross-linking of FcεRI complexes.

In the classical model for allergenic activation, a multivalent antigen – the allergen – enters the body and is captured by a pre-existing complex of an IgE antibody bound to its high-affinity receptor, FcεRI, on the membrane of mast cells, and basophils. Cross-linking of the IgE-FcεRI complex triggers mast cell activation and the release of substances that cause the symptoms of allergy (Figure 1A; Kinet, 1999). Allergic reactions occur within minutes of allergen exposure, differing thereby from other immune responses, which occur on a longer timescale. This is in part due to the unusually high-affinity of the IgE-FcεRI interaction compared to those between other antibody isotypes and their receptors (Gould et al., 2003). Thus allergens bind to IgE-FcεRI complexes formed on these cells in anticipation of exposure to allergen rather than to free IgE, followed by subsequent docking of this complex on unoccupied receptor sites. Only a minuscule proportion of the many receptor molecules on the mast cell membrane need be cross-linked for “immediate hypersensitivity” (MacGlashan, 2005). This mechanism, common to all allergic diseases including allergic asthma, can take place in any bodily tissues populated by IgE and mast cells, notably the respiratory tract, eyes, skin, and gastrointestinal tract, or in the circulation with basophils (systemic anaphylaxis).

Tissue targets are strategically located as barriers against the environment, and the sensitization of mast cells to antigens by antigen-specific IgE antibodies may be one of the many mechanisms, including those of both innate and adaptive immunity that have evolved to impede the entry of pathogens or toxins into the body and thus avert worse mayhem than that generally caused by mere allergic reactions (Galli et al., 1999, 2008; Palm et al., 2012). The central dogma of allergology is that allergen cross-linking of IgE-FcεRI complexes on the mast cells is critical for IgE-mediated mast cell activation. The recent discovery that allergen is not required for certain IgEs to stimulate activities resembling those in the classical model challenges this dogma (Figure 1B). The designation “cytokinergic” is meant to specify both the IgEs displaying such activity and the type of activity itself (Kitaura et al., 2003). The nature of allergen-independent mast cell activation by cytokinergic IgE is unknown.

We review now the experimental evidence for cytokinergic IgE activity and the implications for IgE functions in health and disease. Previous reviews on cytokinergic IgE have focused on the similarities and differences in signal transduction in mast cells by allergens and cytokinergic IgE (Kawakami and Galli, 2002; Kawakami and Kitaura, 2005; Kashiwakura et al., 2011). Here we consider the possible structural determinants of cytokinergic activity in the IgE molecule itself, along with the possible origins and biological function of cytokinergic IgE.

## Cytokinergic IgE Activities

### Effects on FcεRI expression

Two types of cells, blood basophils and mucosal and connective tissue mast cells, are responsible for the immediate hypersensitivity reactions that are the most striking characteristic of the allergic response. Both cell types are generated in the bone marrow, but the latter migrate to the tissues, where they differentiate into mature mast cells expressing the high-affinity IgE receptor, FcεRI (Kitamura and Ito, 2005). This receptor comprises four subunits, one α-chain, containing the IgE binding site in its extracellular sequence, one β-chain and two γ-chains, which are involved in synergistic signal transduction (Kinet, 1999; Turner and Kinet, 1999). Some 35 years ago two groups discovered a positive correlation between FcεRI levels on human basophils and the concentrations of IgE in the serum (Conroy et al., 1977; Stallman and Aalberse, 1977). Several other groups later reached the same conclusion in studies on mast cells (Furuichi et al., 1985; Hsu and MacGlashan, 1996; Yamaguchi et al., 1997). Notably, these experiments required IgE concentrations some 100 times higher (e.g., >1.5 μg/ml; Furuichi et al., 1985) than those that typically suffice for allergen sensitization of mast cells. *In vitro* experiments revealed that the up-regulation of FcεRI is due to the inhibition of endocytosis and under certain circumstances also *de novo* protein synthesis (Yamaguchi et al., 1997). Up-regulation of FcεRI by IgE enhances the IgE-dependent functions of mouse and human mast cells (Galli et al., 1999; MacGlashan, 2005). The effects of IgE binding to FcεRI on a wide range of other cells – antigen-presenting cells such as Langerhans cells, dendritic cells and monocytes, epithelial cells, and muscle cells, all expressing a form of the receptor devoid of the β-chain – are less well documented. That there are other facets of the cytokinergic activity of IgE yet to be explored is suggested by the existence of two other IgE receptors, CD23 and galectin-3 (Liu, 2005), and membrane IgE (the B cell receptor) on IgE-expressing B cells (see Cytokinergic Activity of IgE Via FcεRI in Other Cells or Mediated by Other IgE Receptors).

### Effects on mast cell survival

The incubation of mouse bone-marrow-derived cultured mast cells (BMMC) with certain hybridoma IgEs has the striking effect of increasing cell survival after removal of growth factors (Asai et al., 2001; Kalesnikoff et al., 2001). Implication of IgE binding to FcεRI in this effect was inferred because it was not stimulated by IgG or in BMMC from an FcεRI^−/−^ knockout mouse. BMMC from mouse mutants were also used to exclude any possible cytokinergic activity mediated by alternative IgE receptors, CD23, or galectin-3. Asai et al. (2001) demonstrated that IgE acted by inhibiting apoptosis, rather than stimulating DNA synthesis, while Kalesnikoff et al. (2001) observed up-regulation of Bcl-X_L_, a known anti-apoptotic protein, which may account for this.

These authors noted that the threshold concentration of IgE for stimulation of mast cell survival, as previously found for up-regulation of FcεRI, was several orders of magnitude higher than required for allergen sensitization (Asai et al., 2001; Kalesnikoff et al., 2001). Asai et al. (2001) also observed that washing away the unbound IgE after maximum up-regulation of FcεRI caused an immediate loss of the survival effect, notwithstanding the persistence of the IgE-FcεRI complexes available for antigen activation. We will return to the implications of this observation later (see Is Cytokinergic IgE Activity an Artifact of Adventitious IgE Aggregation? and Dimensional Reduction and Orientation may Drive Self-Association of FcεRI-Bound IgE). Further details relating to IgE regulation of mast cell survival can be found in several reviews (Kawakami and Galli, 2002; Kawakami and Kitaura, 2005; Kashiwakura et al., 2011).

### Effects on mast cell activation

**Figure 2:**
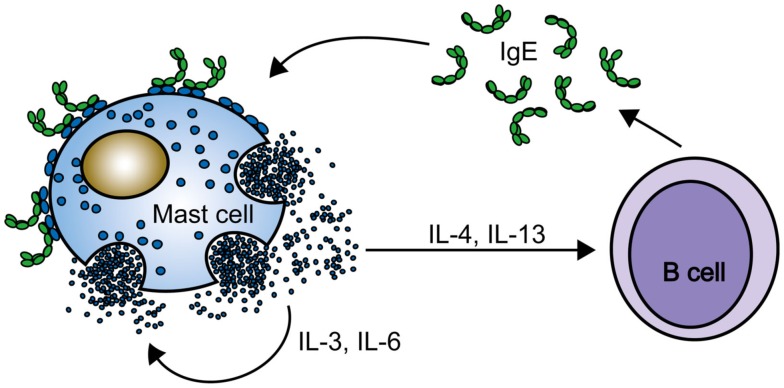
**A positive feedback loop**. Mast cell activation by cytokinergic IgE induces cytokine secretion by mast cells in the absence of antigen. The cytokines stimulate mast cell survival and class switching to IgE in B cells. Continued production of IgE and cytokines occurs in the absence of antigen.

Both publications discussed in the previous section show the results of additional assays of IgE stimulation of cytokine and leukotriene synthesis and secretion and cell degranulation (Asai et al., 2001; Kalesnikoff et al., 2001). Neither group detected leukotriene or histamine release, although these activities were seen in later studies with one of the IgEs, the DNP hapten-specific SPE-7 IgE used by Kalesnikoff et al. (2001). Remarkably, SPE-7 IgE alone stimulated interleukin (IL)-6, TNF-α, IL-4 and IL-13 mRNA, and protein synthesis to a greater extent than antigen (DNP conjugated to human serum albumin; DNP-HSA) binding to the receptor-bound SPE-7. There were significant differences in the kinetics of the response to SPE-7 IgE in the presence and absence of antigen. For example, the IL-6 mRNA and protein expression were delayed and then more prolonged in the absence of antigen. This was also the case for various downstream effects of the IgEs, including phosphorylation of the mitogen-activated kinases, ERK 1/2, p38 and JNK, and the survival-enhancing kinase, PKB. The authors concluded that the cytokines must act in an autocrine or paracrine manner to increase cell survival. Extrapolating to the *in vivo* situation, they pointed out that the cytokines would also act on cell populations other than mast cells to enhance allergic (or cytokinergic) inflammation by, for example, inducing B cells to make more IgE by a positive feedback mechanism (Figure 2). It was not then clear why Asai et al. (2001) failed to observe cytokine secretion or stimulation of signal transduction in their experiments.

### Heterogeneity of IgEs

This discrepancy in the results was soon resolved by comparing the IgEs used in these experiments (Kitaura et al., 2003). Of the eight different hybridoma IgEs examined by Kalesnikoff et al. (2001), three (including SPE-7 IgE) were found to be more “potent” (i.e., cytokinergic) than the other five. In a later survey of several DNP-specific monoclonal IgEs, namely H1 DNP-ε-206, H1 DNP-ε-26 (Liu et al., 1980), and SPE-7 (Eshhar et al., 1980), three commercially available TNP-specific IgEs, IgE-3, C48-2 and C38-2, and an anti-dansyl IgE, 27–34, Kitaura et al. (2003) observed a wide spectrum of effects on mast cell survival and activation. They reproduced their earlier results with the anti-DNP H1 DNP-ε-206 IgE (Asai et al., 2001), which again failed to stimulate cytokine production, and the results of Kalesnikoff et al. (2001) with SPE-7, which not only stimulated IL-6 production, but also led to synthesis/secretion of leukotrienes, histamine release, internalization of IgE, and DNA synthesis. They ranked the IgEs from highly (HC) to poorly cytokinergic (PC) in the order SPE-7 > H1 DNP-ε-26 > H1 DNP-ε-206, according to their various activities and threshold concentrations for these activities. The activity of SPE-7 IgE in stimulating histamine release from BMMC approached that of the same IgE at much lower optimal concentrations, nor was it further increased by the antigen. By contrast, the activity of H1 DNP-ε-206 was comparably stimulated only by antigen. When SPE-7 or H1 DNP-ε-206 IgEs were incubated in media containing varying proportions of wild type BMMC and the FcεRI-deficient FcεRI^−/−^ mutant BMMC, only SPE-7 IgE provoked the wild type cells to secrete cytokines that rescued the FcεRI-deficient cells from apoptosis. Neutralization of IL-3, but not stem cell factor (SCF), ablated this activity in accordance with the later demonstration of IL-3’s importance for mast cell survival in the absence of antigen (Kohno et al., 2005). The differential potency of the IgEs extended to the relevant signaling cascades. Thus, SPE-7 IgE, but not H1 DNP-ε-206 IgE triggers tyrosine phosphorylation and activation of ERK1, ERK2, p38, and Akt (Kitaura et al., 2003). Mice deficient in the kinases implicated in antigen-IgE signal transduction, namely Syk, Btk, and Lyn, were investigated. It transpired that only Syk was required for the stimulation of survival and cytokine synthesis/secretion by cytokinergic IgEs, whereas the full range of HC IgE activities needed the signaling action of the additional kinases.

The same authors tested the effect of injecting IgE- and IgG_2b_-expressing hybridoma cells into mice on the accumulation of mast cells in different bodily locations. The greater accumulation of mast cells in the gastrointestinal tracts of mice injected with H1 DNP-ε-26, rather than with H1 DNP-ε-206 IgE or with the IgG_2b_-secreting hybridoma cells, confirmed the pro-inflammatory activity of HC vs. PC IgE *in vivo*, though without definitive information on the mechanism (see Mechanisms of Cytokinergic Activity).

### Human cytokinergic IgE

The potential implications of cytokinergic IgE for human health and disease prompted the investigation of cytokinergic IgE activity in human mast cells. The first indications came from Gilchrest et al. (2003), who showed that a myeloma IgE alone stimulated I-309 (CCL-1) mRNA expression and release of I-309 protein from human cord blood-derived mast cells (CBMC) cultured with growth factors, but deprived of these before the assay. I-309 (CCL-1) is the ligand for the CCR8 receptor and is responsible for chemoattraction of T helper 2 (Th2)-type T cells, associated with allergic inflammation. The IgE also induced the secretion of GM-CSF and MIP-1α. These results suggest that human IgE, through the production and release of cytokines such as I-309, GM-CSF, and MIP-1α, could promote an inflammatory reaction in the absence of antigen stimulation of the mast cells. Next, Matsuda et al. (2005) showed that a myeloma IgE from the same commercial source as Gilchrest’s induced secretion of IL-8 and monocyte chemoattractant protein 1 (MCP-1) from CBMC. It was found in both studies that addition of IL-4 to the standard culture medium at a late stage before withdrawal of growth factors led to a more robust response (Gilchrest et al., 2003; Matsuda et al., 2005). This can be understood in terms of the effects of IL-4 and IgE on mast cell differentiation and up-regulation of FcεRI (Yamaguchi et al., 1999; Lorentz et al., 2005).

More dramatically, Cruse et al. (2005) showed that a myeloma IgE from a different commercial source than Gilchrest’s and Matsuda’s, stimulated human lung-derived cultured mast cell (HLMC) survival, IL-8 and leukotriene synthesis/secretion, and histamine release and associated Ca^2+^ influx and thus could be described as a human HC IgE. To determine whether this was a consequence of the type of IgE (cf. murine H1 DNP-ε-206 vs. SPE-7 IgE) or the use of HLMC in place of CBMC would require comparison of the different IgEs in the two types of mast cells. The same group later demonstrated that the cytokinergic activity of their IgE depended on the autocrine activity of IL-6 (Cruse et al., 2008), a well-known survival factor for CBMC and LMC (Yanagida et al., 1995; Oskeritzian et al., 2004). Thus, it is clear that certain human IgEs are indeed cytokinergic. Later studies demonstrated that polyclonal human IgEs from atopic dermatitis (AD) patients also exert cytokinergic activities (see Effects of Polyclonal IgEs).

### Effects on mast cell motility, migration, and adhesion

Strikingly, studies of allergy in the mouse model have shown that mast cells can be attracted to HC IgEs *in vitro* and *in vivo* (Kitaura et al., 2005). This was based on a comparison of three IgEs, the “typical” HC SPE-7 IgE and PC IgEs H1 DNP-ε-206 and IgE-E3 in an *in vitro* transwell migration assay. In this technique cells placed in the upper well migrate toward IgE in the lower well. In the case of SPE-7 IgE migration was a function of IgE concentration (between 1 and 30 μg/ml). With H1 DNP-ε-206 and IgE-E3 by contrast, the cells failed to migrate. The kinetics of the migration of the cells toward SPE-7 IgE were similar to those of IgE-sensitized BMMC migrating toward DNP-HSA. This did not occur when BMMC from FcεRIα^−/−^ mice were placed in the upper well. In marked contrast, BMMC from the FcεRIα^−/−^ mice responded vigorously to SCF in control experiments. Wild type BMMC pre-sensitized with PC IgEs also migrated toward DNP-HSA. Migration of BMMC toward the HC IgE was specific to both IgE isotype and antigen. It was necessary to coat the filter between the wells with fibronectin, vitronectin, or laminin, but not by coating with collagen and bovine serum albumin (BSA). Migration was inhibited in the presence of a blocking antibody against integrin β1 (Kitaura et al., 2005). These effects are consistent with the observations on HC IgE induced adhesion and spreading of mouse mast cells on fibronectin-coated plates (Kitaura et al., 2005), and for calcium signaling, actin assembly, and membrane ruffling and spreading of cells of the rat basophilic leukemia cell line (Oka et al., 2004; Pandey et al., 2004).

The corresponding *in vivo* experiments were conducted by epicutaneous application of the same HC and PC IgEs. Skin samples from the mice were stained for mast cells with total and degranulating mast cell numbers counted. The accumulation of mast cells was greater in the mice treated with SPE-7 IgE than with H1 DNP-ε-206 IgE, and similar to the numbers resulting from application of antigen to the same site 24 h after that of H1 DNP-ε-206 IgE.

### Effects of polyclonal IgEs

Experiments with different monoclonal IgEs were central to the discovery of cytokinergic activity. Yet the conditions differed from those in which mast cells encounter polyclonal IgEs in the physiological state. To simulate this situation Kitaura et al. (2003) performed experiments with a mixture of HC and PC monoclonal antibodies, which showed that their cytokinergic activity was simply the average of their individual contributions. To develop a more physiological assay for polyclonal IgE, Kashiwakura et al. (2009) injected mice with a mixture of the house dust mite antigen (*Der f*) and *S. aureus* enterotoxin B (a T cell superantigen) to stimulate a vigorous IgE response, an approach designed to provide a model of AD (Kawakami et al., 2007). Sera from the AD mice, containing ca. 50 μg/ml of IgE, and from wild type controls containing <0.5 μg/ml IgE were added at 10% of total volume to BMMC, and the survival of the cells and cytokine secretion were assayed. Only the AD sera stimulated both survival of the cells and IL-6 and IL-13 secretion. Depletion of IgE from the sera reduced the effects on survival and cytokine secretion; the effects of IgE were not observed with BMMC from FcεRI^−/−^ mice. Using a different model of AD, in which wild type and IgE^−/−^ mice were sensitized by epicutaneous injection of ovalbumin, the authors confirmed that polyclonal IgE promotes mast cell survival and cytokine secretion.

To translate these findings into a human system, Kashiwakura et al. (2009) incubated human CBMC with a 10% v/v dilution of serum from AD patients containing 0.5–12 μg/ml of IgE, or of serum from healthy controls, and measured the secretion of IL-8. The AD sera were significantly more active than non-AD sera and depletion of IgE from AD sera reduced the stimulation of IL-8 secretion to the level attained in non-AD sera. If the polyclonal IgE from the AD patients were a mixture of HC and PC IgE, this would account for the relatively small effects observed in these experiments. However, the concentrations in skin may be sufficient to cause or exacerbate the symptoms of AD.

### Divergent pathways of signal transduction

Kalesnikoff et al. (2001) were the first to point out the difference in the kinetics of mast cell activation by IgE in the presence and absence of antigen (see Effects on Mast Cell Activation). While antigen-triggered mast cell activation is stronger it is of short duration, peaking in minutes, and complete within an hour. By comparison, the activation of mast cells by cytokinergic IgE is delayed and sustained for several hours (Kalesnikoff et al., 2001). It was surmised that the prolonged time course reflected the slow, asynchronous binding of IgE and assembly of the complexes into lipid rafts within the cell membrane. Disruption of the rafts instantly terminated signal transduction. Prolonged stimulation by IgE alone may be required for the *de novo* generation of IL-3 and the survival of mast cells, but weak signaling may fail to drive mast cell degranulation. Simultaneous binding and clustering of the IgE-FcεRI complexes by multivalent allergens may pass the threshold of stimulation required to induce cell degranulation; then the signaling would be rapidly terminated by internalization of the complexes.

Later studies confirmed these observations, clarified the points at which the signaling pathways diverge and identified the resulting downstream events (Charles et al., 2004; Kitaura et al., 2004; Pandey et al., 2004; Sakurai et al., 2004;Yamasaki et al., 2004, 2007; Kohno et al., 2005; Nunomura et al., 2005; Sly et al., 2008). Sly et al. (2008) propose that the weak but sustained ERK phosphorylation in the pro-survival pathway triggers a more prolonged Ca^2+^ influx, associated with the generation of reactive oxygen species (ROS) required for induction of IL-3 synthesis. This is not seen with antigen-dependent stimulation of mast cells. Inhibition of ROS generation with pharmacological inhibitors, e.g., the phospholipase C inhibitor U73122, converts slow to fast kinetics, while the addition of H_2_O_2_ results in IL-3 expression and increased survival of mast cells.

Sly et al. (2008) suggest that local IgE synthesis, regardless of specificity, promotes mast cell survival in mucosal tissues in a positive feedback mechanism (Figure 2). Interestingly, IgE is required to maintain contact sensitivity, although antigen binding to IgE is not involved (Bryce et al., 2004). These authors showed that IgE was not required for the homing of mast cells to mucosal tissues, but was required for the up-regulation of FcεRI on the resident mast cells. Accordingly, the mutual dependence of IgE and FcεRI may represent a homeostatic mechanism in immune defense, associated with the risk of developing allergic disease. Investigations on the signaling pathways of cytokinergic IgE are converging with those on environmental pollution concerned with the effects of oxidative stress in the exacerbation of asthma (Swindle and Metcalfe, 2007; Swindle et al., 2007; Chung and Marwick, 2010; Peden, 2011). Knowledge of the mechanisms involved in the generation of ROS by cytokinergic IgE may contribute to the rational design of future strategies to diminish the impact of pollution (see Germinal Center Reactions and the Local B Cell Repertoire).

## Mechanisms of Cytokinergic Activity

### Is cytokinergic IgE activity an artifact of adventitious IgE aggregation?

We consider first the possibility that has been raised of a trivial explanation for the cytokinergic activity of IgE, based on an adventitious aggregation process. Such a mechanism would imply an intrinsic propensity of IgE to aggregate in solution. Mast cell activation can be nucleated by bringing just two molecules into close proximity and then amplified by more extensive cross-linking to form larger clusters (Segal et al., 1977; Fewtrell and Metzger, 1980; Field et al., 1997). The notion that IgE must be cross-linked by antigen to initiate a signal in mast cells is so firmly entrenched as to lead to a common suspicion that pre-existing aggregates in the IgE preparations must be responsible for cytokinergic activity. That this is not so was painstakingly established by HPLC purification of monomeric IgE in essentially all of the studies. It was further shown that purified aggregates have no activity and that their reconstitution with the monomeric IgE fraction does not increase IgE activity (Kalesnikoff et al., 2001). Neither does prolonged storage or pre-incubation of the IgEs with the BMMC affect their activity. Neither is reversal of the cytokinergic activity by specific monovalent hapten binding (Asai et al., 2001; Pandey et al., 2004) compatible with adventitious IgE aggregation.

### Nature of the “active sites” in cytokinergic IgE

Clustering of HC IgE in the mast cell membrane and signaling through FcεRI implies that complexes with receptor encounter each other directly or indirectly through one or more other molecule(s). How does this occur? The available evidence points to the involvement of contact sites in the N-terminal (Fv) domains of IgE when it is bound to FcεRI on the mast cells. The fact that monoclonal IgEs with different variable regions, regardless of specificity, linked to the same (ε) constant region, exhibited varying degrees of cytokinergic activity implicates this domain. One caveat is that differential glycosylation of PC and HC IgE may vary the exposure of a potential “active site” in the Fc region of IgE. A key role for the Fv domain is also supported by the observation that specific monovalent haptens inhibit the cytokinergic activity, as noted above. The hapten may simply occupy the “active site” or binding may stabilize an alternative conformation of the domain in which the site is occluded or deformed. We enlarge upon the latter possibility below and in Sections “Conformational Isomerism of SPE-7 IgE Facilitates Binding to Structurally Unrelated Antigens” and “Conformational Isomerism as a Basis of Cytokinergic Allergy?”

**Figure 3:**
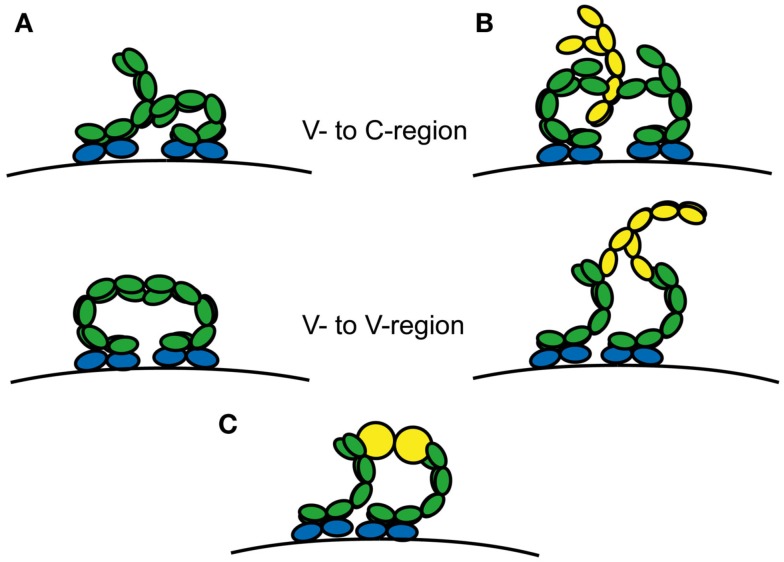
**Alternative schematic mechanisms resulting in cytokinergic cross-linking of IgE-FcεRI complexes**. Complexes are cross-linked **(A)** by receptor-bound IgE Fabs contacting regions of neighboring IgE-FcεRI complexes or **(B)** by bridging with free IgE or **(C)** bridging by other proteins such as HRF or autoantigens. Free IgE and HRF dimer or multivalent autoantigen are shown in yellow.

The rank order of cytokinergic activities of different IgEs was preserved in BMMC tested in serum-free medium (Kitaura et al., 2003). Even in the earlier work of Kalesnikoff et al. (2001) the medium was supplemented solely with BSA and IgE. Thus no extrinsic proteins (other than BSA in some experiments) were present in these systems. The reversal of cytokinergic activity when the unbound IgE is removed from the medium may favor a model in which the free IgE acts as a bridge between two bound IgE molecules (Figure 3B). The participation of both Fv domains of the bound IgE is implied by the necessity to form clusters containing more than two IgE-FcεRI complexes. The rank order of cytokinergic activities may reflect the strength of the IgE-IgE (or IgE-X-IgE) interactions, which is likely to be determined by the highly diverse antigen binding sites in different HC IgEs. The inference is that cytokinergic activity may arise from conformational lability of the Fv domains, allowing exposure of “active sites”. Hapten binding would then stabilize the preferred conformation, resulting in the observed loss of cytokinergic activity. The nature of the interactions has so far proved elusive.

To gain further insight into the structural requirements for cytokinergic activity we constructed recombinant human IgEs with different combinations of V_ε_ and V_L_ sequences. V_ε_ and κ or λ V_L_ cDNA sequences cloned from the nasal mucosa of allergic rhinitis patients (Coker et al., 2003) were used for this purpose. The cytokinergic activities of IgEs with random combinations of V_ε_ and V_L_ were tested in either human peripheral blood-derived cultured mast cells (PBMC; Gould et al., preliminary results) or in CBMC (Kawakami, personal communication) by assaying β-hexosaminidase release (degranulation) and/or IL-8 cytokine secretion. Independent experiments in the two laboratories led to the same conclusion. Thus, a given V_L_ combined with one V_ε_ was highly cytokinergic, while in combination with a different V_ε_ it exhibited only vestigial activity. Likewise, the cytokinergic activity was not consistently associated with the presence of a particular V_ε_ sequence. About one-third of all the recombinant IgEs exhibited a range of activities up to the level observed with mouse SPE-7. These results suggest that the conformation of the whole Fv domain may be important in determining the cytokinergic activity of an IgE; further experiments comparing the cytokinergic activities of recombinant IgEs with natural and scrambled V_ε_ and V_L_ pairs are in progress to resolve this question. In the following sections we review what is known about the conformational properties of antibody Fv regions and how they may impact on cytokinergic activity.

### Conformational isomerism of SPE-7 IgE facilitates binding to structurally unrelated antigens

**Figure 4:**
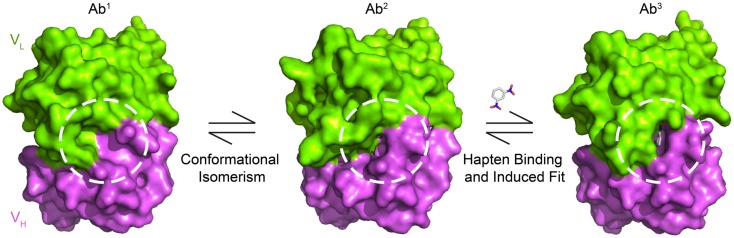
**Conformational changes in SPE-7 alter the shape of the protein surface**. Antigen-free SPE-7 is in equilibrium between two conformers – Ab^1^ (pdb: 1OAQ) that binds proteins and Ab^2^ (pdb: 1OCW) that binds haptens (James et al., 2003). While Ab^1^ has a broad and shallow surface around the key CDRH3 pocket (dashed circle), the pocket is deep, and narrow for Ab^2^. After hapten binding to Ab^2^ a further, induced-fit conformational change occurs resulting in the Ab^3^-hapten bound conformer (pdb: 1OAU).

Antibodies are generated by the adaptive immune system to bind to a seemingly unlimited range of antigens. The initial response occurs through an interaction with a pre-existing polyclonal population of IgM-expressing B cells. The diversity of potential binding specificities is generated by the quasi-random combinatorial recombination of the V, D_H_, and J gene segments encoding the variable regions of the Ig heavy- and light-chains. There is an accompanying contribution to diversity by non-templated nucleotide insertions and deletions at the D_H_-J_H_ junction. However, in practice, the comparatively low number of the body’s circulating primary germline-repertoire B cells (ca. 10^6^) limits this genetic diversity. The extra diversity required for recognition of a wider range of potential antigens originates, it has been suggested, comes from conformational flexibility of the complementarity-determining region (CDR) loops (Stevens et al., 1988; James and Tawfik, 2003a; Thielges et al., 2008). Historically, structural evidence for this conformational diversity within a germline antibody was inferred indirectly from comparisons of the bound and free germline, and affinity-matured, hapten-specific antibodies (Wedemayer et al., 1997; Yin et al., 2003). Recently, Salunke and co-workers have directly shown that germline antibodies can adopt multiple conformations (Khan and Salunke, 2012), with additional diversity conferred by polyreactivity of individual conformers (James and Tawfik, 2003a,b; Sethi et al., 2006). Direct evidence came from kinetic analysis of hapten binding to affinity-matured antibodies (Lancet and Pecht, 1976; Foote and Milstein, 1994; James et al., 2003; Figure 4).

**Figure 5:**
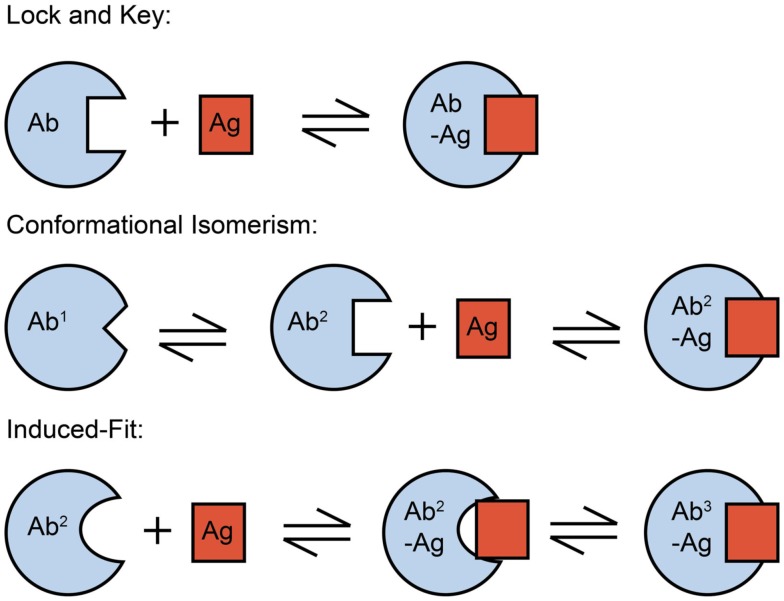
**Mechanism of protein-ligand binding reactions**. An important distinction between the conformational isomerism and induced-fit mechanisms is the step in the binding mechanism at which the conformational change in the protein occurs – before the initial contact with ligand in the case of conformational isomerism, but afterward in the case of the induced-fit mechanism. The functional impact of this difference is that conformational isomerism can drive polyreactivity by providing alternative protein conformers for the binding of different antigens.

Affinity maturation leads to antibodies that bind to their antigen with higher affinity and thus specificity than their germline precursors. This can arise directly through selection of more favorable side chains for antigen binding or indirectly by restricting the conformational flexibility of the CDR loops so as to favor the conformation that the antibody must adopt in its complex with the cognate antigen (Zimmermann et al., 2006, 2010; Thorpe and Brooks, 2007). Indeed, more than 90% of the affinity-matured antibodies studied by Foote and Milstein associate through a single-step mechanism. The pre-rigidification in such a “lock-and-key” mechanism (Figure 5) improves binding by reducing the entropic penalty of freezing out flexible loops (Manivel et al., 2000) and accelerating the association rate constant (Foote and Milstein, 1991). Conformational repositioning can also occur upon antigen binding (Rini et al., 1992) as a result of an “induced-fit” mechanism, whereby an initial low-affinity bimolecular complex forms, which only then undergoes a rearrangement to yield the final high-affinity complex (Figure 5). These changes in the structural dynamics also act to increase antibody specificity.

**Figure 6:**
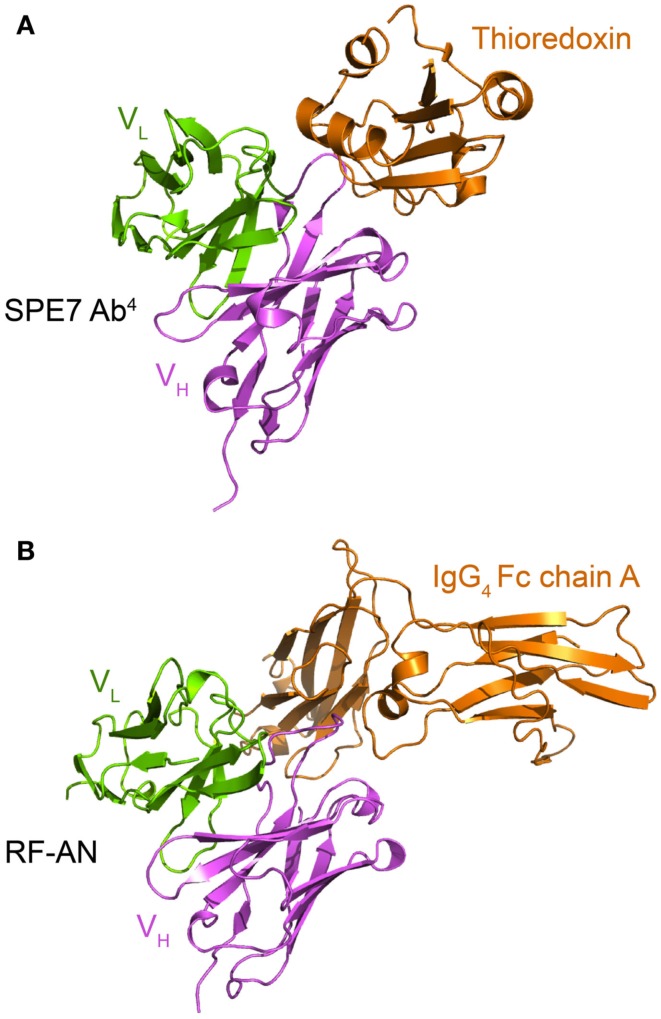
**Both the cytokinergic IgE SPE-7 and the autoantibody RF-AN use similar protein surfaces to mediate cross-reactive protein-protein interactions**. **(A)** Crystal structure of SPE-7 IgE bound to a phage display selected mutant thioredoxin (pdb: 1OAZ). The major SPE-7 IgE conformer (Ab^1^) binds to the thioredoxin to form the Ab^4^-thioredoxin complex (James et al., 2003). **(B)** Crystal structure of the IgM autoantibody RF-AN bound to the IgG_4_ Fc domain (pdb: 1ADQ; Corper et al., 1997). For clarity only the proteins within the asymmetric unit of the crystal and the Fv region of RF-AN are shown.

Even though SPE-7 IgE is a DNP-specific antibody of high-affinity (*K*_d_ = 2 × 10^−8^ M, James et al., 2003), stopped-flow kinetic experiments revealed that the antigen-free state comprises at least, two conformational isomers. Complementary structural studies showed that the preponderant form, Ab^1^ (making up some 80% of the SPE-7 molecules) has a broad and flat conformation that resembles the state in antibody-protein complexes. By contrast, the minor constituent, Ab^2^ (ca. 20% of SPE-7 molecules) has a deep and narrow conformation, reminiscent of antibody-hapten complexes (James et al., 2003; Raghunathan et al., 2012; Figures 4 and 5). The presence of the two conformers enables SPE-7 to bind two types of completely unrelated antigens. Conformer Ab^2^ binds promiscuously to a variety of aromatic ring compounds, with a range of affinities between 20 nM and 100 μM, while the bound state for the more weakly binding compounds essentially resembles the Ab^2^ conformer. Compounds of higher affinity (including the immunizing hapten, DNP-Lys) engender an “induced-fit” rearrangement to a third state, Ab^3^ (James and Tawfik, 2003b, 2005; James et al., 2003; Figure 4). Although a physiological *in vivo* binding partner for the Ab^1^ conformer has yet to be identified, a phage display selected thioredoxin homolog was found to bind to it (Figure 6A), suggesting that such an interaction could occur *in vivo* (James et al., 2003).

The crystal structure of the IgM rheumatoid factor, RF-AN, bound to its autoantigen (IgG_4_ Fc) is instructive in this context (Corper et al., 1997), for it shows that, rather than attaching to conventional antibody-protein-binding CDR loop, it binds at the edge of the loops, leaving much of the usual antigen binding site exposed. While conformational isomerism has not been kinetically demonstrated for RF-AN (Figure 6B), its mode of binding resembles that of the SPE-7 Ab^1^-thioredoxin interaction (Figure 6A). These examples complement each other as for SPE-7 the immunizing antigen is known but not the true *in vivo* cross-reactive protein, and *vice versa* for RF-AN.

### Conformational isomerism as a basis of cytokinergic allergy?

Many of the properties of SPE-7 IgE are shared with other antibodies. Thus, just like the SPE-7 Ab^2^ conformer, many antibodies show cross-reactivity against antigens with similar, though not necessarily identical structure to that of the immunizing antigen. This “molecular mimicry” in fact, plays an important role in allergy (Valenta et al., 1991, 2009; Spitzauer et al., 1994; Aalberse et al., 2001; Schmid-Grendelmeier et al., 2005) and autoimmunity (Rashid and Ebringer, 2012). Moreover, other affinity-matured antibodies have been shown by kinetic criteria to display conformational isomerism; this pertains to as many as 10–20% of those raised against the hapten phenyloxazolone (Lancet and Pecht, 1976; Foote and Milstein, 1994). Further, unrelated antigens have been shown to bind to different conformers of other antibodies (e.g., antibody D1.3 binds to lysozyme and to the antibody E225; Bentley et al., 1990). Therefore, it is highly unlikely that conformational isomerism is unique to SPE-7 IgE, and may be inferred to extend to other IgEs. In the context of cytokinergic IgE activation of mast cells, conformational isomerism may provide a state with the capacity for additional protein-binding interactions. This could be to a third protein, to free IgE, or to another self-protein, thereby bridging two IgE-FcεRI complexes (Figures 3B,C). Alternatively, adjacent IgE-FcεRI complexes may contact each other directly (Figure 3A).

### A different kind of cytokinergic activity: Histamine-releasing factor

Heterogeneity in the cytokinergic function of IgEs immediately brings to mind a similar phenomenon that had already puzzled workers for over 30 years (MacDonald, 1996). Allergic individuals were classified according to two groups, responders or non-responders, depending on the ability of their serum IgE to sensitize mast cells and basophils for the release of histamine, cytokines, and leukotrienes by a protein present in lavage fluids obtained from allergic lesions. IgE^+^ was able to sensitize basophils from both responders and non-responders, whereas IgE^−^ was inactive in the absence of allergen, placing the responsibility firmly on IgE (MacDonald et al., 1987). Histamine-releasing factor (HRF) was purified and the gene cloned, showing its identity with a previously known protein called translationally controlled tumor protein (TCTP), which has a wide range of functions (MacDonald et al., 1995). Thus the obvious candidate as HRF receptor appeared to be IgE^+^ itself, but the first attempts to demonstrate direct binding to IgE were unsuccessful (Wantke et al., 1999).

This may have been due to unfavorable experimental conditions (perhaps the limited range of IgEs or the assay conditions). Nevertheless, in early 2012 binding of recombinant mouse HRF to certain mouse monoclonal IgE^+^ and IgG^+^ preparations and to polyclonal mouse IgE^+^ was demonstrated (Kashiwakura et al., 2012a). The use of a large panel of monoclonal Igs together with BMMC revealed that recombinant HRF bound with varying strength to about one-third of the Igs, and that potency of the IgEs in assays of HRF-induced airway inflammation was roughly correlated with this binding activity. Injection of the HRF into mice of different genetic deficiencies showed that the airway inflammation is dependent on mast cells, B cells, FcεRI, and the FcR γ-chain. Monoclonal IgE^+^, but not IgE^−^ induced passive cutaneous anaphylaxis in mice, mediated by FcεRI - a further demonstration of *in vivo* activity.

The structural properties of IgE^+^ were investigated. Their Fabs were as active in binding HRF as intact IgE molecules, and binding was inhibited by antigen, implying that HRF binds at a site in the variable regions. Interestingly, there appeared to be a relation to the presence of κ-chains derived from two particular members of the κ germline gene family (8–30 and 2–137), paired with heavy-chains from different V_H_ families. Recombinant HRF was also split into smaller fragments, two of which inhibited HRF activities *in vitro* and *in vivo* in mice. The activity of the corresponding HRF peptides in the human system remains to be evaluated; clinical applications may be envisaged.

The native HRF is a dimer with two disulfide bonds uniting the subunits. Cysteine to alanine mutations or reduction of the disulfide bonds eliminated the activity in IgE^+^-mediated BMMC or peritoneal mast cell activation (Kashiwakura et al., 2012a). These authors propose a model to explain how dimeric HRF molecules are able to cross-link IgE-FcεRI complexes to activate the mast cell (Figure S8 in Kashiwakura et al., 2012a). The presence of two different inhibitory sequences in HRF implies the existence of two different contact sites in each IgE^+^ Fv. A simplified form of the complex, utilizing one Fv in each of two adjacent IgE-FcεRI complexes and only one of the contact sites in each subunit of the HRF dimer is represented in Figure 3C. It is easy to envisage how such a complex might nucleate larger clusters through the vacant contact sites to form an effective signaling platform for mast cell activation.

The lack of cytokinergic activity of IgE^+^, even at high concentrations, in the absence of HRF, and the lack of HRF responsiveness of SPE-7 IgE and other HC IgEs (at low concentrations) show that the two activities are not equivalent (Xie et al., 2008; Kashiwakura et al., 2012a). However, neither HC IgE nor IgE^+^ require the presence of an antigen, and therefore both may be considered as “cytokinergic IgEs” according to the original definition (Kitaura et al., 2003).

The examples here of HC and IgE^+^ illustrate the potential for multiple mechanisms of cytokinergic IgE activity. Both HC IgE and IgE^+^ are evidently involved in allergic inflammation in the absence of allergen. Both are implicated in the exacerbation of atopic asthma and allergy. Cytokinergic IgE activity may provide a rationale for the paradoxical observation that the risk of developing an “allergic disease” and severity of the disease is more closely related to the levels of total IgE than allergen-specific IgE in serum (Burrows et al., 1989; Sunyer et al., 1996; Beeh et al., 2000). It may also account for “allergic disease” in non-atopic patients, in whom the usual skin prick tests for allergen sensitivity and serum titers of allergen-specific IgE are negative, but who present with the usual signs and symptoms of allergic disease (see Germinal Center Reactions and the Local B Cell Repertoire).

### Cytokinergic activity of IgE via FcεRI in other cells or mediated by other IgE receptors

Cells other than mast cells and basophils express FcεRI (the “high-affinity” IgE receptor) and many types of cells express CD23 (the “low-affinity” IgE receptor). FcεRI is expressed in Langerhans cells, dendritic cells, monocytes, smooth muscle cells, and epithelial cells, while CD23 is expressed in many of these cell types and also in B cells, T cells, and follicular dendritic cells (Gould et al., 2003; Gould and Sutton, 2008). There are two splice variants of human CD23, CD23a, and CD23b, differing in 6/7 amino acids at the cytoplasmic N-terminus; these are expressed in different circumstances and have different functions (Gould et al., 2003; Gould and Sutton, 2008). Galectin-3, also known as Mac-1 and IgE binding factor (Huff et al., 1983), binds to both IgE and FcεRI, amongst other ligands (Liu, 2005). Early experiments with BMMC from mice in which the genes were inactivated argue against a role for CD23 or galectin-3 in mediating cytokinergic IgE activity (see Effects on Mast Cell Survival).

A recent study suggests that macrophages may exert cytokinergic IgE activity through FcεRI in an important physiological context – atherosclerosis (Wang et al., 2011). Serum IgE levels were elevated in two groups of Chinese patients with myocardial infarction or unstable angina pectoris. IgE and FcεRI were observed in atherosclerotic lesions and localized to the majority of macrophages and a minority of smooth muscle cells and epithelial cells in the tissue. A fat diet was used to develop a mouse model of atherosclerosis. The disease was attenuated in FcεRI^−/−^ and TLR^−/−^ mice suggesting synergistic activity between these receptors. IgE in the range of 6–100 μg/ml stimulated signal transduction and cytokine secretion by isolated macrophages from the mice and SPE-7 IgE was more potent than H1 DNP-ε-206 IgE. Weaker IgE responses could be induced in the smooth muscle cells and epithelial cells. The large discrepancy in the concentration of IgE required to activate mast cells and macrophages may reflect the relatively low levels of receptor expression or absence of the signal amplifier β-chain in these cells. Since there are few B cells in the tissue, IgE must be imported from the circulation. It is certainly questionable whether local IgE concentrations in heart tissue ever reach the levels required for macrophage activation, although heart mast cell involvement in cardiac disease is by now well established (Genovese et al., 2010).

No activities of HC IgE in IgE-expressing B cells have come to light. SPE-7 IgE hybridoma cells appear to grow normally and IgE levels in the culture media rise well above the concentrations required for cytokinergic activity in mast cells. High concentrations of the FcεRI and the topology of HC IgE-FcεRI complexes in the membrane of mast cells (and other FcεRI bearing cells) may be critical for cytokinergic IgE activity (see Fab Pre-Orientation in the IgE-FcεRI Complex on the Mast Cell Membrane). In so far as mast cells are implicated in diseases other than allergy, e.g., autoimmunity, HIV infection, and malaria, cytokinergic IgE may have a wider significance in disease pathogenesis.

### Secondary receptor editing as a source of cytokinergic IgEs

A cost/benefit balance is implicit in the possession of a large antibody-directed repertoire while precluding antibody targeting of self-antigens (Ehrlich’s *horror autotoxicus*; Ehrlich, 1900). Induction of tolerance to self-antigens is inseparable from the prevention of autoantibody formation, given that after V(D_H_)J recombination in the bone marrow up to 75% of B cells are self-reactive (Wardemann et al., 2003; Nemazee, 2006). During B cell development within bone marrow, tolerance is achieved through a combination of deletion of self-reactive B cells and receptor editing. The editing involves exchange of the V-segment attached to DJ-Cμ for the heavy-chain, or of the J-Cκ or J-Cλ for the light-chain, thereby altering the B cell receptor specificity, so that it no longer recognizes the self-antigen. It is now clear (Eisenberg, 2012) that, after release from the bone marrow, B cells that have migrated to peripheral lymphoid tissue can undergo further rounds of receptor editing, called “receptor revision.” On the one hand, this may serve to eliminate self-reactive B cells that escape from the bone marrow or are generated by somatic hypermutation in the periphery. On the other, this process may generate polyspecific, autoreactive B cells anew, either before or after switching to IgE, that may exert cytokinergic activity. Studies on circulating B cells have shown that a significant proportion (∼5%) produce polyspecific IgM and IgG (Wardemann et al., 2003; Nemazee, 2006), of which a proportion may be generated in the periphery.

The sequence of events in receptor editing and revision is itself well characterized. The Th2 inflammatory cytokine IL-6 induces the expression of RAG1 and RAG2, products of the recombination activating genes *rag1* and *rag2*. A complex of RAG1 and RAG2 can itself initiate the process of receptor revision, since all the other machinery for repairing DNA breaks in cells is constitutive (Nemazee, 2006). RAG genes are normally silenced once a functional Ig is expressed in B cells, but may be re-expressed in the presence of IL-6 (Hillion et al., 2007a,b). IL-6 secretion is commonly observed when mast cells are stimulated by cytokinergic IgE; when this occurs in the target organs of allergy, it may lead to the local expression of polyspecific/cytokinergic IgE.

Polyspecific IgE is well characterized in at least two pathological conditions, parasitic worm infection, and chronic sinusitis with polyps (ChRwP). Helminths induce Th2-type immune responses and regulatory T cells that produce anti-inflammatory cytokines. This is accompanied by the synthesis of abundant polyspecific IgE, little or none of which is specific for the parasite antigens (Harnett and Harnett, 2010). This appears to be the parasite’s strategy to evade detection and elimination by the host immune system. Similarly, nasal polyps in ChRwP are characterized by the presence of abundant Th2 cytokines, regulatory T cells, and polyspecific IgE. Compelling evidence supports the hypothesis that *S. aureus* infection causes this polyposis, yet only small proportions of the IgE are directed against the bacterial proteins and allergens (Zhang et al., 2011). The high concentrations of polyspecific IgE may well exert cytokinergic activity, contributing to chronic inflammation and allowing *S. aureus* to exist in the commensal state. Re-expression of RAG1 and RAG2 in the nasal B cells and receptor revision may be instrumental in generating the polyspecific IgE to disarm the immune response.

### Structural impact of secondary editing on Fv properties

After affinity maturation of binding to a specific antigen, residues from the three CDR loops on the heavy- or light-chains, and often both, directly contact the antigen, an event in which CDRH3 often plays a dominant role (Stanfield et al., 1993). CDRH3 lies close to the V_H_-V_L_ domain interface; the relative orientation of the domains changes on antigen binding (Stanfield et al., 1993; Teplyakov et al., 2011), and also in the course of affinity maturation, thus enhancing the affinity of antigen binding (Chatellier et al., 1996), as discussed in Section “Conformational Isomerism of SPE-7 IgE Facilitates Binding to Structurally Unrelated Antigens.” The relative orientations of the V_H_-V_L_ domains vary considerably; thus one analysis of the crystal structures shows that their angular disposition varies between −60.8° and −31.0° (Abhinandan and Martin, 2010). Mutations at the V_H_-V_L_ interface also affect the structural stability of the Fv region (Chatellier et al., 1996; Vargas-Madrazo and Paz-Garcia, 2003; Nakanishi et al., 2008; Caravella et al., 2010). It appears then that after receptor revision the new V_H_-V_L_ may well be less stable than the original Fv, thus alteration of the V_H_-V_L_ pairings could increase the conformational isomerism enabling antibodies to interact with themselves (self-association) or other proteins (Figure 3). Mutations at particular positions in the V_H_ interface with V_L_ have been used to create single chain antibodies, similar to those expressed in camels (Barthelemy et al., 2008). Certain V_H_-V_L_ combinations may actually result in domain swapping between IgEs bound to adjacent FcεRI on mast cells, or between the bound and free IgEs (see below).

### Dimensional reduction and orientation may drive self-association of FcεRI-bound IgE

**Figure 7:**
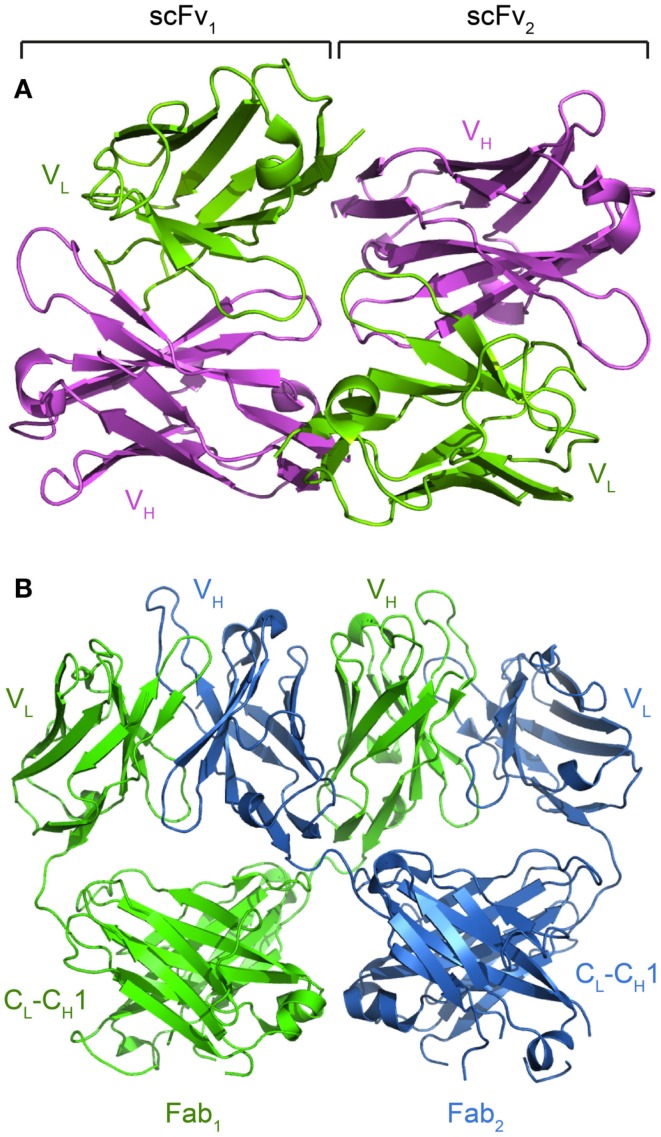
**Crystal structures reveal alternative modes of antibody self-interactions**. **(A)** V_H_-V_L_ interactions are seen for the scFv of MFE-23 (pdb: 1QOK; Boehm et al., 2000). **(B)** V_H_ domain swapping between the anti-HIV Fab 2G12 (pdb: 1OM3; Calarese et al., 2003).

The evidence so far available suggests that cytokinergic activity is related to receptor clustering on the surface of mast cells, and that HC IgEs induce more extensive clustering than PC IgEs (Kalesnikoff et al., 2001; Kitaura et al., 2003). This process must depend in large measure on the enormous increase in binding affinity that results when the reactants are confined in the plane of the membrane, by reason of their high concentration in two dimensions and their much-reduced orientational freedom (see e.g., DeLisi (1981) who calculates a reduction by a factor of about 10^5^ M in *K*_d_ relative to the same reactants in free solution). The likelihood therefore is that, in the normally attainable concentration range, interaction between the molecules we are considering would be undetectably low in solution. Schweitzer-Stenner and Pecht (2005) have modeled the situation for varying numbers of FcεRI molecules per cell. With typical numbers, e.g., 3 × 10^5^ molecules/cell, the probability of productive collisions would be extremely high. Thus, given the mobility of receptors in the membrane, the ability of IgEs to self-associate after they bind to FcεRI, is plausible. There is moreover ample evidence for Fab-mediated self-association of antibodies at high local concentrations; thus, Shire and co-workers have used the anti-IgE omalizumab as a case study for such a phenomenon (Yadav et al., 2012). This could occur, for example, through the variable region on one Fab contacting the variable or constant region on another (Figure 3A). A range of potential orientation modes for self-association has been observed crystallographically, with the most extreme case being domain swapping (Boehm et al., 2000; Lee et al., 2002; Calarese et al., 2003; Igawa et al., 2010; Figure 7).

### Fab pre-orientation in the IgE-FcεRI complex on the mast cell membrane

**Figure 8:**
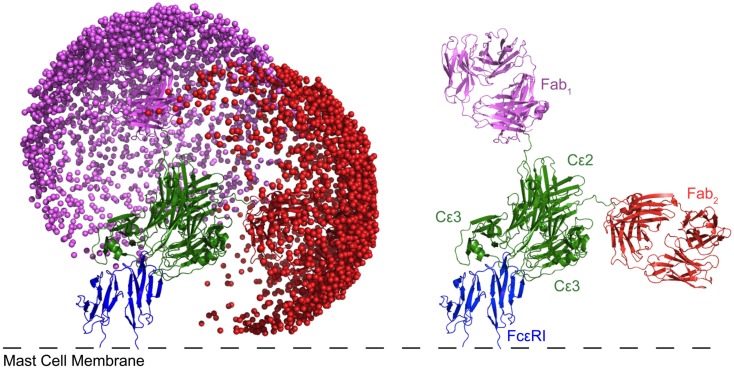
**Modeling of IgE bound to FcεRI reveals that one of the Fabs predominately lies parallel to the membrane**. Each sphere represents a possible position of the antigen binding site of the Fab. Cε4 is present, but obscured by Cε2 and 3. The modeled structure not only accounts for the observed restricted mobility of Fabs on IgE relative to IgG, but also suggests that two neighboring IgE-FcεRI complexes are pre-disposed for ready cross-linking either by self-association or through a bridging protein. For more details of modeling see Hunt et al. (2012).

IgE possesses unique properties that may pre-dispose it to cytokinergic activity. The high-affinity of binding (*K*_d_ ∼ 10^−10^ M) and slow dissociation (*t*_1/2_ ∼ 14 days in tissue) imply that IgE is effectively permanently attached to FcεRI on the cell membrane. IgE has one more pair of Ig domains in its Fc region than IgG, which causes IgE to adopt an asymmetrically bent structure (Wan et al., 2002; Hunt et al., 2012). The bend becomes more acute on binding to FcεRI (Holdom et al., 2011; Hunt et al., 2012), causing the extra (Cε2) domains to orient the Fab domains away from the membrane, thus giving less obstructed access to the antigen (Figure 8). We surmise that the angular freedom of the Fabs allows the IgE to accept a wider range of antigens. Structural freedom may, on the other hand, also promote interactions with each other or other molecules to promote cytokinergic activity. We suggest that this structurally predetermined orientation of the Fabs may play an important role in predisposing the mast cells and other cells expressing FcεRI to cytokinergic IgE activation.

Activation of mast cells in the allergic response is initiated by antigen-induced cross-linking of cell surface IgE-FcεRI. The optimum distance between the antigen epitopes was measured with rigid bivalent allergens (DNP-modified oligodeoxynucleotides), showing that there needs to be sufficient proximity of the cross-linked complexes (<70 Å apart) for significant stimulation of degranulation, although signaling upstream of the Ca^2+^ response was unimpaired with greater distances between the epitopes (Paar et al., 2002). The crystal structures of two allergens each bound to two IgE antibody Fabs show an approximately linear structure (Li et al., 2008), compatible with the presentation of the Fabs oriented perpendicular to the membrane when IgE binds to FcεRI on the mast cell (Niemi et al., 2007). Pre-orientation of the IgE Fabs would also allow self-interaction or bridging, e.g., by free IgE or HRF (Figure 3).

### Most HC IgEs have polyreactivity to autoantigens

Human IgEs do not bind to mouse FcεRI, but mouse IgE does bind to human FcεRI (Conrad et al., 1983). Conveniently, this allows mouse IgEs, as well as human IgEs, to be tested for polyreactivity in human FcεRI-expressing cells. To examine the relationship between cytokinergic IgE activity and polyreactivity Kashiwakura et al. (2012b) tested a number of well characterized mouse and human HC and PC IgEs for binding to a panel of autoantigens routinely used for the diagnosis of autoimmunity (single- and double-stranded DNA, thyroglobulin, β-galactosidase) and HRF. They also confirmed the cytokinergic activity of two of the human IgEs (Matsuda et al., 2005) and compared the cytokinergic activity of sera containing polyclonal IgE from AD patients and healthy controls in CBMC.

These experiments demonstrated that mouse HC but not PC IgEs, and the human monoclonal IgEs that exhibited cytokinergic activity in the earlier study, recognized one or more of the autoantigens. Sera from AD patients but not the healthy controls showed increased reactivity against the single- and double-stranded DNA and β-galactosidase and increased levels of HRF. Sera from AD patients with DNA-reactive IgE induced more IL-8 secretion from CBMC than sera from healthy controls. The individual HC IgEs bound to different subsets of these autoantigens, showing that there are multiple modes of IgE-FcεRI self-association responsible for cytokinergic IgE activity, consistent with the heterogeneity of cytokinergic IgEs based on the strength of signal transduction (see Heterogeneity of IgEs). This is the result that would be expected if cytokinergic IgE activity arises from the random association of heavy- and light-chains after VD_H_J recombination (cf. Wardemann et al., 2003), receptor editing, or receptor revision and switching to IgE.

Many previous studies have revealed the existence of IgE autoantibodies (see Conformational Isomerism as a Basis of Cytokinergic Allergy?). Some of these are known to mediate mast cell activation in the classical manner with autoantigens (Valenta et al., 2009). The new insight offered by Kashiwakura et al. (2012b) is that there may be a cytokinergic element in IgE autoimmunity.

## Possible Origins and Biological Significance of Cytokinergic IgE Activities

### Germinal center reactions and the local B cell repertoire

An alluring conjecture is that HC IgEs could be created *de novo* in the target organs of inflammation, caused by allergens or alternatively by non-allergic mechanisms, such as viral or bacterial infections. The resulting inflammatory cytokines may in turn induce polyclonal B cell proliferation and lymphoid neogenesis; i.e., the formation of tertiary lymphoid tissue, promoting germinal center reactions. The location of these events, determined by the cytokine environment, may specify the antibody class by directing class switching, e.g., to IgG in the synovial tissue in rheumatoid arthritis or IgE in the respiratory tract mucosa in rhinitis and asthma. Lymphoid neogenesis is well recognized in chronic autoimmune diseases, chronic infections, and B cell malignancies (Aloisi and Pujol-Borrell, 2006). It also occurs normally in the gastrointestinal tract, which is under constant bombardment from a complex and ever-changing mixture of bacterial products (Spencer et al., 2009). It has received less attention in relation to allergic disease, where IgE is the relevant antibody class, and to which we will confine our discussion.

By germinal center reactions we mean those that cause changes in the sequence of the expressed Ig genes in B cells secondary to the original VD_H_J recombinatorial diversity created in bone marrow. This takes its name from the classical germinal centers located in secondary lymphoid tissue. The three germinal center reactions in B cells are somatic hypermutation, receptor revision, and class switch recombination. Somatic hypermutation introduces point mutations in the variable regions of the Ig heavy- and light-chains, which may either increase or decrease the affinity for an antigen and serves as the basis for affinity maturation in the immune response. In the mechanism of class switch recombination, the expressed variable region gene is recombined with one of the downstream constant region genes in the tandem array in the Ig heavy-chain locus on chromosome 14 and this diversifies the effector function and anatomical distribution of the antibody. Receptor revision is perhaps the most dramatic event, since it almost inevitably leads the B cell to lose its original antigen specificity or at least its affinity, but also offers a small chance of acquiring a new specificity against an environmental antigen. We suggest that randomization of the heavy- and light-chain combination in IgE is the origin of cytokinergic IgE (see Secondary Receptor Editing as a Source of Cytokinergic IgEs).

There is ample evidence for the induction of tertiary lymphoid tissue and germinal center reactions in the respiratory tract in allergic disease. Local somatic hypermutation is revealed by the presence of clonal families in the nasal mucosa in rhinitis (Coker et al., 2003, 2005) and in the bronchial mucosa in asthma (Snow et al., 1995, 1997, 1998). Receptor revision is supported by the presence of RAG1 and RAG2 proteins in the tissue and the altered B cell repertoire (Coker et al., 2005). The abundance of Th2 and mast cells secreting IL-4, IL-13, and IL-6 provides an ideal environment for receptor revision and local class switching to IgE. Class switch recombination to IgE is experimentally observed by the presence of activation-induced cytidine deaminase in the tissue, along with ε germline gene transcripts, ε switch circle transcripts, ε-chain mRNA, and members of the same clonal family in IgE and IgA (Cameron et al., 2003; Coker et al., 2003;Takhar et al., 2005, 2007; Gould et al., 2006). The observed outcome is a massive increase in the proportion of IgE-expressing B cells (1/24), and of the plasma cells (1/8) in the nasal mucosa in rhinitis, compared to 1/10,000 of IgE-expressing B cells in the circulation (KleinJan et al., 2000). In atopic rhinitis allergen-specific IgE produced by B cells in the nasal mucosa forms a substantial minority of the total RNA, and invariably a greater proportion than it represents in the circulation (Smurthwaite et al., 2001). We have estimated that the rate of IgE antibody synthesis in the nasal mucosa is 100 times greater than that required to saturate FcεRI on mast cells in the tissue (Gould et al., 2003). Therefore the local IgE concentrations must be sufficient for cytokinergic activity. IgE in the circulation may simply be the spill over from respiratory tract and other tissue compartments (Gould et al., 2006; Eckl-Dorna et al., 2012).

Germinal center reactions are transient in secondary lymphoid tissue because they feed on the antigens that are eliminated in the immune response. Yet one cannot exclude the possibility that the target organs of allergic disease provide a survival niche for IgE-expressing B cells and plasma cells (Luger et al., 2009). In principle, this could be due in part to the cooperation between the B cells that continue to secrete IgE that binds to neighboring mast cells that respond to the IgE, according to the positive feedback mechanism illustrated in Figure 2. Persistent production of HC IgEs in the tissue may thus lead to the symptoms of chronic disease in the absence of allergen. Preliminary evidence showing the activity of cytokinergic IgEs produced by the randomization of ε heavy- and light-chains in recombinant IgEs derived from the nasal mucosa of rhinitis patients (see Nature of the “Active Sites” in Cytokinergic IgE) is fully consistent with this scenario.

In summary we suggest the following pathway via cytokinergic IgE to chronic disease:

Primary (viral, bacterial, allergic) inflammation in the target organs of allergy → somatic hypermutation, receptor revision, and class switching to IgE → IgEs with instability in Fv due to random combination of V_H_ and V_L_ sequences during receptor revision → polyspecific cytokinergic IgE → chronic disease.

What then might be the biological function of local receptor revision and the production of polyspecific cytokinergic IgE in the respiratory tract and gastrointestinal mucosa and skin? All these are barrier tissues involved in immune defense against multitudinous ever-changing external threats. It may be vital to maintain the diversity of the local antibody repertoire (Spencer et al., 2009), and this is surely an effective, but risky strategy.

### Diagnosis and therapy

IgE is central to allergic disease but as we have indicated above IgEs are heterogeneous and similar symptoms are generated by different pathomechanisms. Cytokineric IgEs may be produced in an inflammatory response and may cause the symptoms of allergy in the absence of allergen. The non-anaphylactogenic anti-IgE antibody, omalizumab, has proved to be a generally effective therapy in the clinic for moderate to severe asthma and in a wide variety of pre-clinical investigations on other allergic conditions. Since omalizumab acts by competing with FcεRI for IgE binding it does not prevent IgE synthesis. Therefore, intensive efforts have been devoted to do this. For example, IgEs have been engineered to target the extra-membrane-proximal domain of IgE on B cells to induce cell death (Feichtner et al., 2008; Brightbill et al., 2010; Chen et al., 2010). The murine precursor of omalizumab has been re-engineered to increase its affinity for IgE and induce the co-ligation of membrane IgE and FcγRIIb, which inhibits IgE synthesis, as well as neutralizing free IgE (Chu et al., 2012). It has been lamented recently that both anti-IgE therapies and the more commonly prescribed anti-inflammatory corticosteroids have actually been “too successful,” in that they benefit patients with different underlying pathobiologies (Drazen, 2012). A better understanding of these pathobiologies would enable more specific diagnostic tests and targeted treatments.

We have attempted in this review to alert the reader to the different modes of IgE activity in the activation of mast cells and basophils. Inhibitors of oxidative stress (see Divergent Pathways of Signal Transduction) and of HRF binding to IgE (see A Different Kind of Cytokinergic Activity: Histamine-Releasing Factor) have been flagged up as potential therapies to intervene in the activity of cytokinergic IgE, but further research will undoubtedly uncover others.

## Conclusion and Unanswered Questions

We hope we may have persuaded the reader that cytokinergic activity is no mere academic curiosity, relevant only to model hapten-specific mouse antibodies acting on cultured mast cells, but that it is likely also to trouble humans. Most provocative perhaps may be the conjecture that these cytokinergic IgEs play a part in triggering and perpetuating the symptoms of allergic rhinitis and asthma. Therefore, this seems to us an area of no small topical interest.

If it be granted that cytokinergic activity is a real immunological phenomenon, then the immediate challenges in understanding its molecular basis are mainly structural, biochemical, and biophysical. The conjectural mechanisms we have outlined of the events that follow the binding of a cytokinergic IgE to the mast cell need to be tested. Detailed structural and dynamic studies have so far been confined to a single case (SPE-7 IgE), and need to be extended to other cytokinergic IgEs to establish the generality of the conclusions. Nor has anything yet been undertaken to relate the highly variable level of cytokinergic activity to amino acid sequence differences. Additional questions also arise: are differences in cytokinergic IgE potency linked to degree of conformational mutability? Are the structural and dynamic characteristics of the IgEs governed to any significant extent by the V_H_ and V_L_ pairings? Are the V_H_ and V_L_ sequences and pairings in IgE in the target organs of allergy unique to these sites, inasmuch as they are probably generated *de novo* by local germinal center reactions? What is the evolutionary advantage of cytokinergic IgEs? Considering their apparent place in the front-line of immune defense, do they perhaps perform an innate immune function, rather like the circulating IgM produced by naïve B cells? Do cytokinergic IgEs also pose a risk of engendering a “pseudo-allergy” and, if so, how might one intervene to promote or suppress their formation? The answers to many of these questions are well within the reach of present-day technology.

## Conflict of Interest Statement

The authors declare that the research was conducted in the absence of any commercial or financial relationships that could be construed as a potential conflict of interest.
